# Chlorambucil-Induced Acute Interstitial Pneumonitis

**DOI:** 10.1155/2014/575417

**Published:** 2014-02-12

**Authors:** Hammad Shafqat, Adam J. Olszewski

**Affiliations:** ^1^Memorial Hospital of Rhode Island, 111 Brewster Street, Pawtucket, RI 02860, USA; ^2^The Warren Alpert Medical School of Brown University, Providence, RI 02912, USA

## Abstract

Chlorambucil is an alkylating agent commonly used in treatment of chronic lymphocytic leukemia (CLL). We report a case of interstitial pneumonitis developing in an 83-year-old man 1.5 months after completing a six-month course of chlorambucil for CLL. The interstitial pneumonitis responded to therapy with prednisone. We performed a systematic review of literature and identified 13 other case reports of chlorambucil-induced pulmonary toxicity, particularly interstitial pneumonitis. No unifying risk factor could be discerned and the mechanism of injury remains unknown. In contrast, major randomized trials of chlorambucil therapy in CLL have not reported interstitial pneumonitis as an adverse effect, which may be due to the rarity of the phenomenon or due to underreporting of events occurring after completion of treatment. Clinicians should consider drug-induced interstitial pneumonitis in the differential diagnosis of a suggestive syndrome developing even after discontinuation of chlorambucil.

## 1. Introduction

Chlorambucil is an alkylating agent used for treatment of indolent lymphoproliferative disorders, particularly chronic lymphocytic leukemia (CLL) [1]. It is a relatively well-tolerated drug with myelosuppression constituting its principal toxicity [2]. Other alkylating agents such as busulfan and cyclophosphamide have been implicated in toxic lung injury [3]. Data regarding chlorambucil-induced lung injury is however very limited and consists of scattered case reports. Pulmonary toxicity has been reported as a dose-independent adverse effect of chlorambucil occurring during or after discontinuation of the therapy. We report a case of chlorambucil-induced interstitial pneumonitis along with a systematic review of literature summarizing the evidence of lung toxicity as a rare adverse effect of the agent.

## 2. Case Report

**Figure 1:**
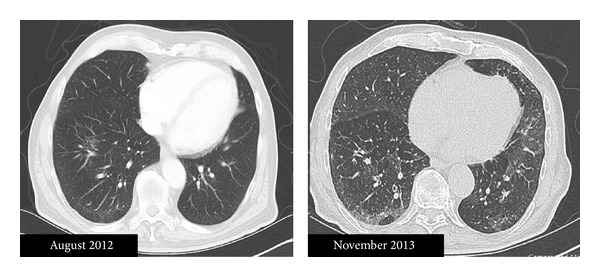
Computed tomography image of the patient's lungs prior to treatment with chlorambucil (August 2012) and after diagnosis of pneumonitis (high-resolution scan, November 2013), demonstrating interval development of interlobular septal thickening more prominent in the periphery of the right lung, suggestive of interstitial lung disease.

An 83-year-old man presented to our hospital with acute onset of dyspnea and hypoxia. The patient had been diagnosed with CLL 14 months prior to the event, with asymptomatic lymphocytosis of 15.5 × 10^9^/L, evidence of CD5, CD23, and CD38-positive, CD10-negative B-cell leukemia on flow cytometry, hemoglobin of 133 g/L, and platelet count of 132 × 10^9^/L. After 8 months of watchful waiting he experienced progressive adenopathy and thrombocytopenia, which constituted indications for therapy. The patient received single-agent oral chlorambucil at the dose of 0.6 mg/kg every 14 days for 6 months. There were no significant toxicities during therapy, which resulted in a partial remission of CLL and resolution of adenopathy and cytopenias. Six weeks after the last dose of chlorambucil, the patient noted rapidly escalating dyspnea on exertion and then resting shortness of breath. There was minimal nonproductive cough and no fever, hemoptysis, or chest pain. The patient had a history of 60 pack-year tobacco use but had quit smoking over 5 years ago and had no known underlying lung disease. His medical history was significant for diabetes mellitus, hypertension, benign prostatic hyperplasia, glaucoma, and gout. Ambulatory medications included metformin, doxazosin, and latanoprost. On admission the patient was tachypneic and hypoxic with a resting pulse oximetry of 85% on 2 liters of oxygen but without fever, rales, or wheezing on examination. The neutrophil count was normal (5.3 × 10^9^/L, reference range 2.0–10.0 × 10^9^/L) as were serum brain natriuretic peptide (44 pg/mL, reference range <100 pg/mL), lactate dehydrogenase (116 units/L, reference range: 98–192 units/L), urine *Streptococcus pneumoniae *and *Legionella pneumophila* antigen tests, and cytomegalovirus IgG and IgM serologies. A 2D echocardiogram revealed normal systolic and diastolic function without any significant valvular dysfunction or pericardial effusion. A computerized tomography (CT) angiogram of the chest on admission ruled out a pulmonary embolism but demonstrated bilateral basal interlobular septal thickening compatible with interstitial pneumonitis. A staging CT scan performed 1 year earlier did not show any evidence of interstitial pneumonitis (Figure 1). No infectious cause was found for the patient's symptoms and there was no improvement with azithromycin. The patient declined a diagnostic open lung biopsy, and a clinical diagnosis of drug-induced interstitial pneumonitis was made. He was treated empirically with prednisone 1 mg/kg and albuterol inhalers which led to a rapid resolution of his dyspnea and hypoxia. Prednisone was tapered off over 2 months. Pulmonary function tests performed after discontinuation of steroids revealed the ratio of forced expiratory volume in 1 second to forced vital capacity (FEV1/FVC) of 73%, vital capacity of 2.63 L (74% predicted), total lung capacity of 5.23 L (121% predicted), and diffusion capacity for carbon monoxide, hemoglobin corrected, of 19.29 mL/min/mmHg (96% predicted), interpreted as consistent with normal aging process. Although the patient's respiratory function returned to baseline, a repeat high-resolution CT scan 3 months later showed persistent subpleural fibrosis.

## 3. Systematic Review

We performed a literature search in the MEDLINE database using keywords “chlorambucil AND (pneumonitis OR pulmonary fibrosis).” The initial query resulted in 76 articles. Only 13 articles describing case reports or case series of drug-induced pulmonary toxicity were included for analysis (Table 1) [4–16]. Review articles or those reporting other pulmonary diseases including infectious pneumonias were excluded. In order to evaluate possible risk factors for chlorambucil-induced interstitial pneumonitis, full text of identified reports was scrutinized to extract data on patient demographics, diagnostic modality, chlorambucil dosage and treatment duration, and outcomes. We also reviewed randomized controlled trials using chlorambucil in CLL published in the past 10 years to collect rates of reported lung toxicity.

Pulmonary side effects of long-term chlorambucil therapy were initially reported by Rubio in 1972, with a number of subsequent cases, mostly in patients with CLL. In some cases, the presentation of lung disease was acute, occurring immediately after ingestion of the alkylating agents, while in others symptom developed weeks to months of therapy or after its discontinuation. Some patients treated solely with supportive care died because of respiratory failure, but most of those treated with steroids and discontinuation of chlorambucil recovered. In many cases the diagnosis of interstitial lung fibrosis was confirmed by an open or transbronchial lung biopsy, without evidence of an opportunistic infection or leukemic infiltration. One report showed pathology consistent with bronchiolitis obliterans organizing pneumonia.

Although the identified case reports provide a link between interstitial pneumonitis and chlorambucil exposure, this has not been observed in clinical trials of the drug. Among 777 patients receiving first-line therapy for CLL with chlorambucil alone or fludarabine with or without cyclophosphamide in the British LRF-CLL4 trial, there were no reports of drug-induced pneumonitis [17]. Similarly, the toxicity was not reported in the American Intergroup trial (*N* = 509) and the German CLL Study Group trial of chlorambucil against fludarabine (*N* = 193) or in the international randomized trials of chlorambucil against alemtuzumab (*N* = 297) and bendamustine (*N* = 319) [2, 18–20]. In all those studies the main side effect of chlorambucil was bone marrow suppression (20–30%) while only <5% of patients developed cough. Increased rates of noninfectious pulmonary toxicity have not been noted in trials utilizing the combination of chlorambucil with the monoclonal antibody rituximab either [21, 22].

## 4. Discussion

Chlorambucil was one of the first effective chemotherapeutic agents employed for treatment of CLL and other indolent lymphoproliferative disorders. Although it showed inferior response rates and progression-free survival compared with newer agents, randomized trials showed no evident disadvantage in overall survival. Moreover, there was no difference in either progression-free or overall survival between chlorambucil and fludarabine in patients older than 65 years [18]. Because purine analogues are associated with higher rates of infectious complications, chlorambucil remained an acceptable treatment option for older or frail patients with CLL [23, 24]. Most recently, this paradigm changed when improved survival in older CLL population was achieved by combining chlorambucil with CD20-directed antibodies rituximab and obinutuzumab [25]. Chlorambucil-based chemoimmunotherapy is thus likely to become a widely used first-line treatment standard for older patients with CLL.

Our report and prior literature reveal interstitial pneumonitis as a rare and potentially late-onset adverse effect of chlorambucil. The events may range from acute interstitial pneumonitis to chronic and occasionally fatal lung fibrosis and occur weeks to months after discontinuation of the drug, with no clear dose-response relationship. Although in our case only clinical and radiographic data supported drug-related etiology of the pneumonitis, the findings meet the diagnostic criteria for “possible usual interstitial pneumonia,” defined as subpleural, basal predominance of reticular abnormalities without honeycombing and with lack of inconsistent features [26]. Recent guidelines recognize clinical history and high-resolution CT imaging as sufficient for diagnosis of idiopathic pulmonary fibrosis, although a surgical lung biopsy may still be needed to differentiate the usual interstitial pneumonia from nonspecific interstitial pneumonia and chronic hypersensitivity pneumonitis [27]. Serum markers such as KL-6 and surfactant proteins SP-A and SP-D may be elevated in a proportion of patients with drug-induced pneumonitis, but they are not available for general clinical use [28]. In older patients the diagnosis may need to rely on the CT imaging and exclusion of infectious causes using serology or bronchoalveolar lavage. Although we could not formulate firm recommendations for management based on case reports, a recent review of pneumonitis induced by antineoplastic agents recommended high-dose methylprednisolone (1 gram daily for 3 days) for patients with respiratory failure and lower doses (methylprednisolone 60 mg every 6 hours) for less severe cases [29]. The optimal duration of steroid therapy is uncertain.

The pathogenesis of chlorambucil-induced lung toxicity has not been specifically studied. Mechanisms of injury with other alkylating agents include generation of reactive oxygen species and alteration in alveolar repair mechanisms [28]. Cyclophosphamide has been reported to cause two separate patterns of toxicity: early-onset pneumonitis, responsive to steroids, and late-onset fibrosis with progressive, refractory course culminating in death [30]. In animal models melphalan exposure leads to a burst of proinflammatory interleukins, subsequent lung infiltration by neutrophils within 24 hours, and then a sustained lymphocytic response over days to weeks resulting in lung fibrosis [31]. Early treatment with steroids reduced alkylator-induced airway inflammation and collagen deposition in a mouse model [32]. Our review did not reveal a pattern of factors that would suggest specific risk for chlorambucil-induced interstitial pneumonitis, other than male predominance. Tobacco use, the main risk factor for idiopathic pulmonary fibrosis, was mentioned in two reports and our patient had extensive, albeit distant smoking history. Only one article indicated an underlying hypogammaglobulinemia and receipt of immunoglobulin supplementation. A genetic risk factor for chlorambucil-induced interstitial pneumonitis is possible considering multiple polymorphisms predisposing to idiopathic pulmonary fibrosis, including surfactant proteins A2 and C, as well as telomerase mutations [33–35]. Recently, a polymorphism in the promoter of *MUC5B*, the gene encoding mucin 5B, has also been associated with interstitial lung disease regardless of smoking history [36].

In conclusion, since chlorambucil is likely to remain an important agent for the treatment of CLL in older patients, clinicians should be aware of the potential for rare interstitial pneumonitis induced by this agent. Infectious causes of respiratory illness will always predominate in CLL due to immune suppression characteristic of this disease, but patients who are receiving or who recently completed chlorambucil therapy should be evaluated for possible alkylator-induced lung toxicity if they present with characteristic clinical and radiographic findings. Prompt discontinuation of chemotherapy and initiation of steroids may be life-saving. Future research is needed to identify clinical and genetic predisposing factors for this toxicity.

## Figures and Tables

**Table 1:** Reports of chlorambucil-induced interstitial lung disease.

Report, year	Clinical information	Diagnosis of pneumonitis	Cumulative chlorambucil dose	Duration of therapy (months)	Treatment and outcome
Rubio 1972 [4]	Four patients CLL Tobacco: NR	OLB in 2 cases	NR	NR	NR All expired
Rose 1975 [5]	67-year-old female CLL Tobacco: NR	NR	NR	15	NR
Refvem 1977 [6]	60-year-old male CLL Tobacco: NR	Autopsy: alveolar epitheliolysis and fibrosis	2500 mg + prednisolone	~9	Multiple antibiotics Expired
Cole et al. 1978 [7]	60-year-old female polycythemia vera Tobacco: none	OLB: interstitial fibrosis	4130 mg	22	Methylprednisolone Improved
Godard et al. 1979 [8]	67-year-old male CLL Tobacco: 30 py	OLB: interstitial pneumonitis	7500 mg	36	Improved
Lane et al. 1981 [9]	74-year-old black male CLL Tobacco: NR	OLB: focal interstitial fibrosis	161 mg + prednisone	6	Prednisone Expired
Carr 1986 [10]	62-year-old white male CLL Tobacco: NR	OLB: interstitial fibrosis	4300 mg	56	Prednisone Improved
Giles et al. 1990 [11]	67-year-old male CLL Tobacco: NR	Transbronchial biopsy: desquamative interstitial pneumonitis	2016 mg + prednisone	44	Expired
Mohr et al. 1993 [12]	57-year-old male CLL Tobacco: NR	BAL	640 mg	6	Prednisone, IVIG Improved
Crestani et al. 1994 [13]	48-year-old male CLL Tobacco: none	BAL	8340 mg	44	Improved
Khong and McCarthy 1998 [14]	77-year-old male CLL Tobacco: 45 py	OLB: interstitial fibrosis	2700 mg	60	Prednisone Improved
Tomlinson et al. 1999 [15]	70-year-old male B-cell lymphoma Tobacco: NR	Transbronchial biopsy: organizing pneumonia	200 mg + mitoxantrone and prednisone	1.5	Prednisolone Improved (Rechallenged with chlorambucil without recurrence)
	56-year-old female B-cell lymphoma Tobacco: NR	OLB: interstitial infiltrates with eosinophils	600 mg + mitoxantrone and prednisone	6	Improved
Kalambokis et al. 2004 [16]	70-year-old male CLL Tobacco: NR	OLB: bronchiolitis obliterans organizing pneumonia	200 mg + mehtylprednisolone	1.5	Improved
Current report	83-year-old male CLL Tobacco: 60 py	CT scan only	456 mg	6	Prednisone Improved

Abbreviations: BAL: bronchoalveolar lavage; IVIG: intravenous immunoglobulin; NR: not recorded; OLB: open lung biopsy; py: pack-year.
